# Complement C1s deficiency in a male Caucasian patient with systemic lupus erythematosus: a case report

**DOI:** 10.3389/fimmu.2023.1257525

**Published:** 2024-02-26

**Authors:** Jessica S. Kleer, Lillemor Skattum, Denise Dubler, Ingeborg Fischer, Armin Zgraggen, Esther Mundwiler, Min Jeong Kim, Marten Trendelenburg

**Affiliations:** ^1^ Laboratory of Clinical Immunology, Department of Biomedicine, University of Basel, Basel, Switzerland; ^2^ Division of Internal Medicine, University Hospital, Basel, Switzerland; ^3^ Department of Laboratory Medicine, Section of Microbiology, Immunology and Glycobiology, Lund University, and Clinical Immunology and Transfusion Medicine, Region Skåne, Lund, Sweden; ^4^ Division of Pathology, Cantonal Hospital Aarau, Aarau, Switzerland; ^5^ Division of Rheumatology, Cantonal Hospital Aarau, Aarau, Switzerland; ^6^ Division of Laboratory Medicine, Cantonal Hospital Aarau, Aarau, Switzerland; ^7^ Division of Nephrology , Cantonal Hospital Aarau, Aarau, Switzerland

**Keywords:** immunodeficiency diseases, complement C1s, non-sense mutation, systemic lupus erythematosus (SLE), lupus nephritis (LN)

## Abstract

Deficiencies of the early complement components of the classical pathway (CP) are well-documented in association with systemic lupus erythematosus (SLE) or SLE-like syndromes and severe pyogenic infections. Among these, complete C1s deficiency has been reported in nine cases so far. Here, we describe a 34-year-old male patient who presented with severe, recurrent infections since childhood, including meningitides with pneumococci and meningococci, erysipelas, subcutaneous abscess, and recurrent infections of the upper airways. The patient also exhibited adult-onset SLE, meeting 7/11 of the ACR criteria and 34 of the 2019 EULAR/ACR classification criteria, along with class IV-G (A) proliferative lupus nephritis (LN). A screening of the complement cascade showed immeasurably low CH50, while the alternative pathway (AP) function was normal. Subsequent determination of complement components revealed undetectable C1s with low levels of C1r and C1q, normal C3, and slightly elevated C4 and C2 concentrations. The patient had no anti-C1q antibodies. Renal biopsy showed class IV-G (A) LN with complement C1q positivity along the glomerular basement membranes (GBMs) and weak deposition of IgG, IgM, and complement C3 and C4 in the mesangium and GBM. In an ELISA-based functional assay determining C4d deposition, the patient’s absent complement activity was fully restored by adding C1s. The genome of the patient was analyzed by whole genome sequencing showing two truncating variants in the *C1S* gene. One mutation was located at nucleotide 514 in exon 5, caused by a nucleotide substitution from G to T, resulting in a nonsense mutation from Gly172 (p.Gly172*). The other mutation was located at nucleotide 750 in exon 7, where C was replaced by a G, resulting in a nonsense mutation from Tyr250 (p.Tyr250*). Both mutations create a premature stop codon and have not previously been reported in the literature. These genetic findings, combined with the absence of C1s in the circulation, strongly suggest a compound heterozygote C1s deficiency in our patient, without additional defect within the complement cascade. As in a previous C1s deficiency case, the patient responded well to rituximab. The present case highlights unanswered questions regarding the CP’s role in SLE etiopathogenesis.

## 1 Introduction

The complement system (CS) is a collection of over 50 soluble and membrane-bound proteins that play an important role in host defense and removal of apoptotic cells and immune complexes (ICs). It can be activated via the classical (CP), lectin (LP), and alternative pathways (AP) (1). The CP is activated when C1q binds to ICs, apoptotic bodies, or pathogen-associated patterns. C1q forms a pentameric complex with the Ca^2+^-dependent C1s-C1r-C1r-C1s tetramer that binds to the collagen-like region of C1q. Binding of C1q to activator structures results in the activation of C1r. Activated C1r then cleaves and activates C1s which in turn cleaves C4 and C2 to form the CP C3 convertase (C4b2a) (2).

Systemic lupus erythematosus (SLE) is a systemic autoimmune disease of unknown cause. It is characterized by a dysregulated immune system, resulting in the generation of IgG autoantibodies to numerous self-antigens. The source of autoantigens is probably apoptotic cells (3). IgG-containing ICs activate the CS, which leads to tissue injury, e.g., glomerulonephritis (GN). Interestingly, individuals with a deficiency of early proteins of the CP have a greater risk of developing SLE (4), which represents a paradox since the CS is an important effector of inflammation that mediates the pathogenesis of SLE (5).

The strength of the association of a complement deficiency with SLE increases from C2 (10% prevalence) over C1r/s (57% prevalence), C4 (75% prevalence) to C1q (90% prevalence) (4). The mechanism underlying this association has not yet been established. The cases of SLE associated with complement deficiency are rare and only account for a tiny minority of all patients with SLE. However, they may provide an important clue to the etiology of the disease (6). Excluding the present case, selective complete C1s deficiency has been reported in only nine cases so far (7–13). The *C1S* gene is located on the short arm of chromosome 12 in the region p13 and contains 12 exons (14). Genetic analyses have shown that the disease is inherited in an autosomal recessive mode (9–11). However, the penetrance and expressivity are currently undetermined due to the low number of cases. Four of the reported cases had symptoms of SLE of which three cases had renal diseases (7, 11, 13). Two patients carrying a compound heterozygote mutation with one allele encoding p.Glu579* displayed unique symptoms including fever of unknown origin (FUO), convulsions, and disturbances of consciousness (9, 12). Three out of the reported cases were asymptomatic (11, 12). In the present study, we report a case of selective C1s deficiency resulting from a compound heterozygosity for two different abnormalities in the *C1S* gene, which have not been described so far.

## 2 Case description

**Figure 1:**
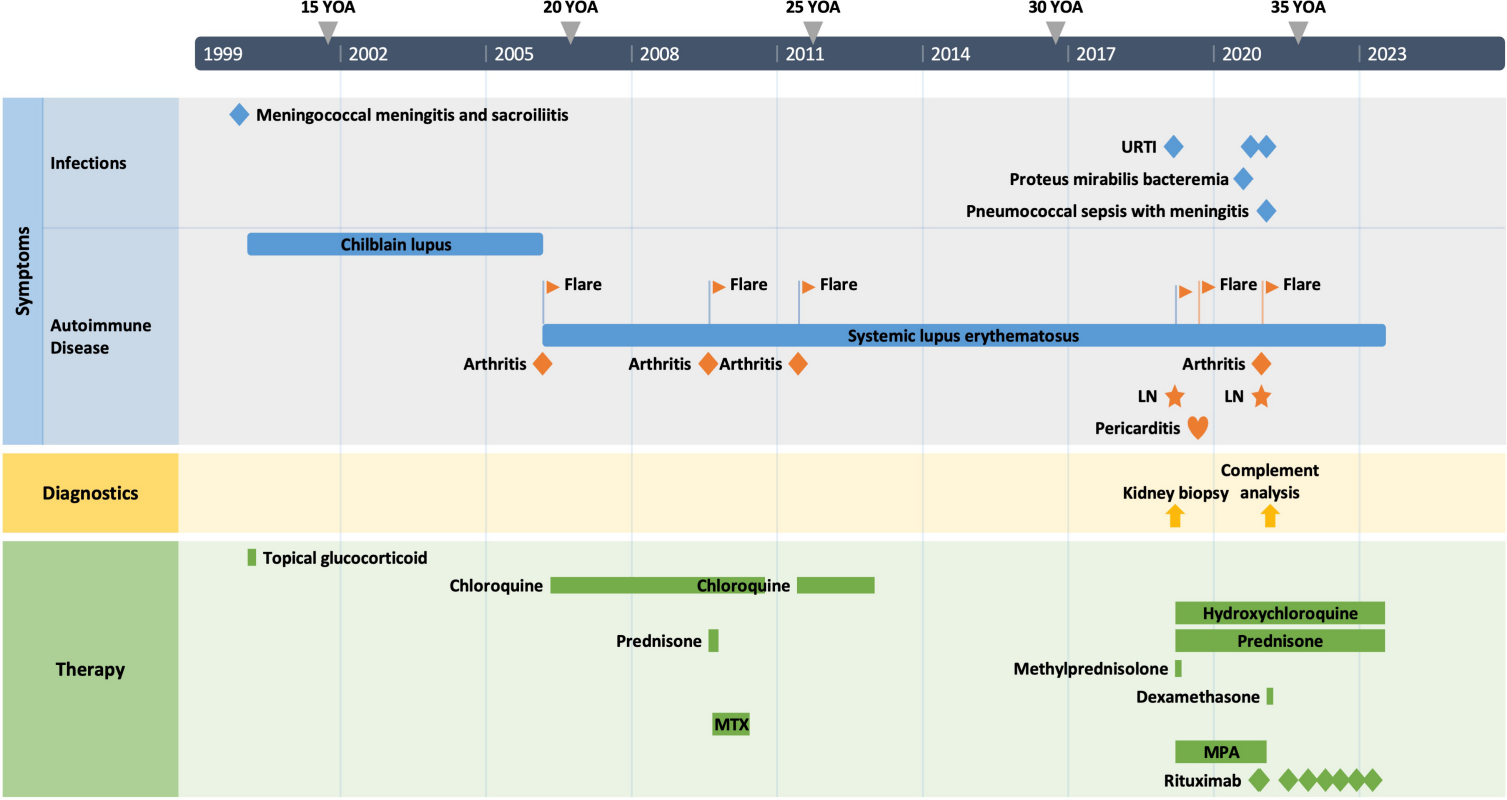
The patient’s clinical course. YOA, years of age; URTI, upper respiratory tract infection; LN, lupus nephritis; MTX, methotrexate; MPA, mycophenolic acid.

**Figure 2:**
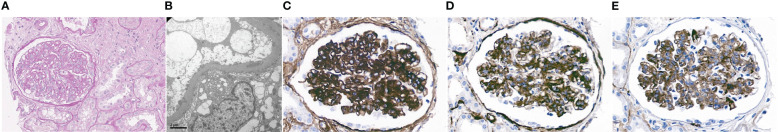
Microscopy study of kidney biopsy. **(A)** Periodic acid–Schiff staining (magnification ×40) disclosed global mesangial hypercellularity and matrix expansion. **(B)** Electron microscopy (EM) showed subendothelial deposits. **(C)** Immunohistochemistry revealed deposition of C1q, **(D)** C5-9, and, to a lesser degree, **(E)** IgM in the mesangium and along the glomerular basement membrane.

**Figure 3:**
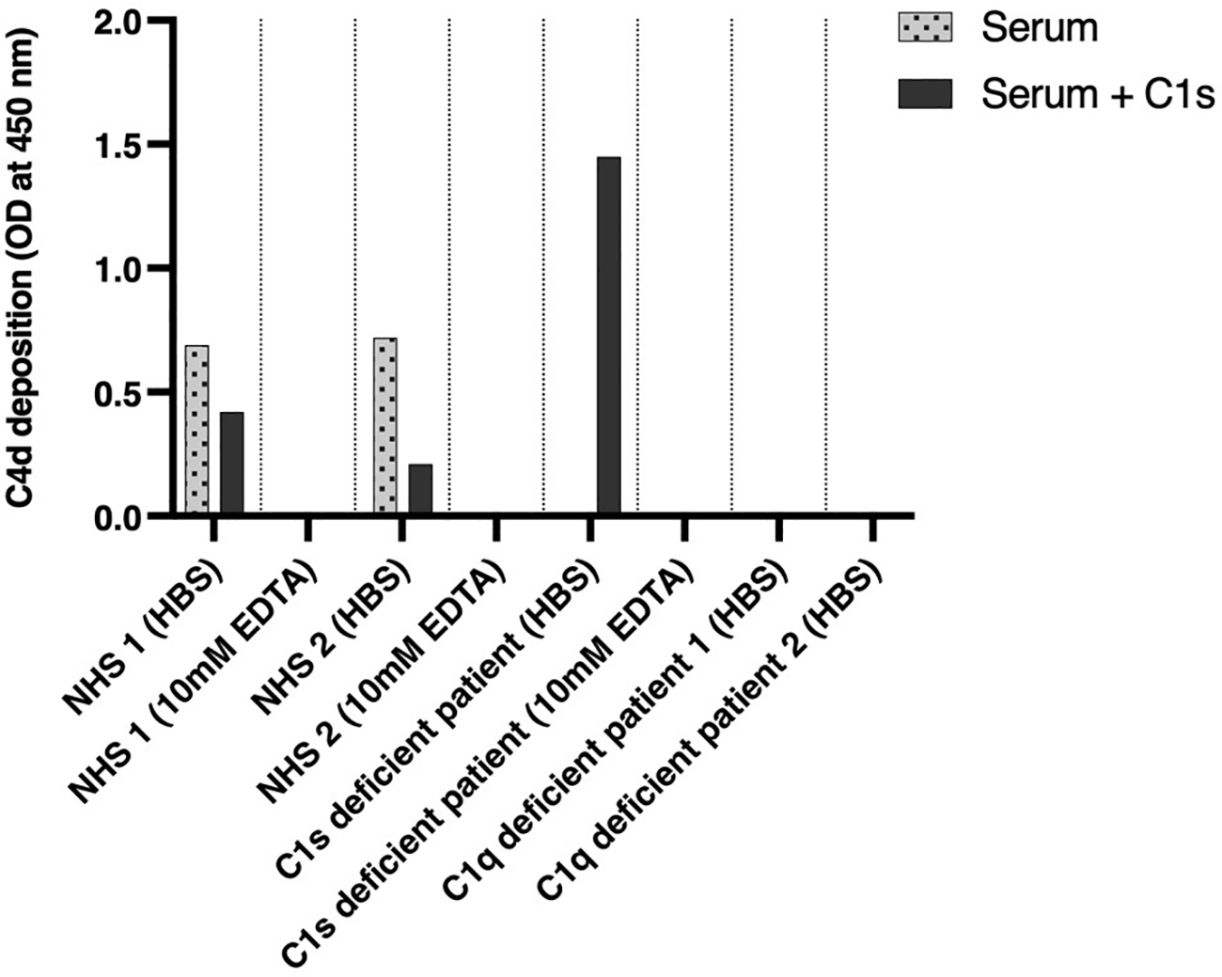
Functional complement assays and C1s reconstitution. Two healthy donors, two C1q-negative patients, and a C1s-negative patient were investigated for the activation of the classical complement pathway by an ELISA-based assay determining C4d deposition (dotted bars). Complement activation was investigated again after substitution of C1s proenzyme (dark gray bars). NHS, normal human serum.

The patient is a 34-year-old male Caucasian who presented recurrent infections and SLE manifestations. His symptoms started at the age of 13 years and are illustrated in **Figure 1**. He was born into a non-consanguineous marriage and has three healthy older sisters. Except for diabetes mellitus type 2 of his father, the family history was unremarkable. At the age of 13 years, he was hospitalized with meningococcal meningitis and sacroiliitis with *Neisseria meningitidis* type Y:4:P1.5. At that time, the AP was normal at 131%, whereas the CP was less than 13 units (26–46 U). Complement deficiency was suspected but not further investigated. However, he was vaccinated with diphtheria–tetanus–acellular pertussis (DTaP), *Haemophilus influenzae* type b (Hib), pneumococcal polysaccharide, and meningococcal polysaccharide vaccine. Two years later, the patient presented with cold fingers and toes with bluish-livid discoloration and scaling. Antibodies against SSA were detected. The diagnosis of chilblain lupus was made and therapy with topical glucocorticoids was initiated. At 19 years of age, the patient reported worsening of skin lesions, malar rash, photosensitivity, fever, malaise, arthralgias, and loss of appetite and weight. Antinuclear antibodies (ANA) were elevated, with a titer of 1:1,280, and exhibited a fine speckled pattern on Hep-2 cells (AC-4). Anti-double-stranded DNA (anti-dsDNA) antibodies were within the normal range. At that time, he fulfilled 4 of the 11 American College of Rheumatology (ACR) revised criteria for the classification of SLE (15) and had a score of 12 using the 2019 EULAR/ACR classification criteria for SLE (16). The chilblain lupus was considered to have evolved into SLE, and therapy with chloroquine 200 mg was started. Three years later, he had a flair with oligoarthritis. Laboratory tests revealed positive rheumatoid factor (RF), and the extractable nuclear antigen (ENA) panel detected positive anti-SS-A/Ro60, anti-SS-B, and anti-Sm. The established treatment was extended with prednisone and methotrexate (MTX). Prednisone was tapered, MTX was later discontinued due to malaise, and chloroquine was discontinued due to reduced drug compliance. Because of another flair with oligoarthritis at the age of 24, chloroquine was started again but not continued for drug malcompliance. At the age of 32, the patient presented with flank pain, macrohematuria, and hypertension which had occurred after an upper respiratory infection. The physical examination was otherwise unremarkable. Laboratory results showed macrohematuria, proteinuria of 1.3 g/24 h, and elevated creatinine (280 µmol/L) with limited GFR according to CKD-EPI of 25 ml/min/1.73 m^2^. A complete blood count revealed normocytic anemia. ANA were still positive at 1/1,280 and the anti-dsDNA level was now found to be slightly elevated, falling within the low-positive range [233 IU/ml (<200 IU/ml)]. The urinary sediment showed non-glomerular hematuria. A kidney biopsy was performed, allowing the visualization of nine glomeruli (**Figure 2**). By light microscopy, six out of nine glomeruli showed mesangial hypercellularity and matrix expansion as well as focal low-grade thickened glomerular basement membranes. Electron microscopy revealed subendothelial deposits. Immunofluorescence (IF) showed granular deposition of IgG, IgM, and C3 on glomerular basement membranes and in the mesangium. Immunohistochemically, deposition of C5-9 and strong deposition of C1q could be detected. In contrast, the deposits of C3, C4, IgG, and IgM were weak in immunohistochemistry. According to the International Society of Nephrology/Renal Pathology Society 2003 classification, this renal biopsy finding was classified as lupus nephritis (LN) class IV-G (A). The patient was treated with hydroxychloroquine (HCQ), prednisolone, and mycophenolate mofetil (MMF). Half a year later, the patient was hospitalized because of acute pericarditis, whereupon therapy with HCQ and prednisolone was increased. In August 2020, the patient had bacteremia with *Proteus mirabilis* based on inguinal erysipelas. Approximately one and a half years after the diagnosis of lupus nephritis, the patient had increasing activity of LN with hematuria and proteinuria. Therefore, an additional rituximab infusion was initiated in late 2020. In February 2021, he developed pneumococcal sepsis with meningitis. Full complement diagnostic was performed and the results are shown in **Table 1**. The functionality of the classical pathway of complement as determined by CH50 was below the lower limit of detection, whereas the functional activity of the AP was normal. C1s was not measurably low, while C1q and C1r were only decreased. The levels of the C4, C2, and C1 inhibitors were elevated. The patient did not have anti-C1q antibodies. The suspected diagnosis of C1s deficiency was confirmed by C1 reconstitution experiments, in which externally added C1s could restore the complement activation in the patient’s serum (**Figure 3**). The diagnosis was confirmed by whole genome sequencing, which was performed in Lund/SE. A compound heterozygosity was detected, consisting of two novel variants in the *C1S* gene. One mutation was located at nucleotide 514 in exon 5, caused by a nucleotide substitution from guanine to thymine, resulting in a nonsense mutation at codon 172. The other mutation was located at nucleotide 750 in exon 7, where cytosine was replaced by guanine, resulting in a nonsense mutation at codon 250. Both mutations create a premature stop codon. Due to the severe infection, MMF was stopped and prednisone was reduced as much as possible to 5 mg per day. Further reduction of prednisone led to an increase in the patient’s skin manifestations. Thus, in analogy to the case of LN in C1s deficiency published in 2010 by Bienaimé et al. (13), the patient was treated with rituximab 1 g every 4 months, under which no severe infections or lupus flares have occurred to date. The renal function has stabilized with an eGFR of 80 ml/min/1.73 m^2^ within the usual range of variation. Proteinuria remains stable at approximately 0.5 g/day.

**Table 1:** Analysis of complement parameters and autoantibodies.

Analyses (Lund)	Result	Ref. interval	Unit	Analyses (Basel)	Result	Ref. interval	Unit
AP, hemolytic assay	Normal function						
CP, hemolytic assay	Lost function						
AP, ELISA	62	30–113	%				
CP, ELISA	<1	69–129	%	CH50	<5	>68	%
C1q	40	78–131	%	C1q	0.08	0.14–0.35	g/L
C1 inhibitor	0.63	0.20–0.38	g/L				
				C3	0.96	0.90–1.80	g/L
				C4	0.44	0.10–0.40	g/L
C2	164	77–159	%	C2	0.044	0.019–0.043	g/L
C1r	23	71–133	%				
C1s	<5	72–146	%				
C1r plasma	33	78–131	%				
				**Autoantibodies**			
				ANA-titer^a^	640	<80	
				ACA	<40	<80	
				Anti-dsDNA	<9.8	<27	IU/ml
				Anti-C1q	4	<20	U/ml
				Anti-SS-A/Ro60	>1,300	<20	Units
				Anti-SS-B	518	<20	Units
				Anti-RNP/Sm	250	<20	Units
				Anti-Sm	76	<20	Units

CP, classical pathway; AP, alternative pathway; ANA, antinuclear antibodies; ACA, anticytoplasmatic antibodies.

a Indirect immunofluorescence assay (IIFA) on HEp-2 cells showed fine speckled and large/coarse speckled patterns (AC-4/-5).

## 3 Diagnostic assessment

### 3.1 Material and methods

#### 3.1.1 Functional complement assays

In Lund/SE, functional activity of the CP and AP was assessed via hemolysis-in-gel assays according to Truedsson L et al. (17) and the Wieslab Complement System Classical Pathway ELISA (COMPLCP310) and Wieslab Complement System Alternative Pathway ELISA (COMPLAP330) (Svar Life Science, Malmö, Sweden). In Basel/CH, MicroVue CH50 Eq EIA from Quidel (San Diego, CA, USA) was used to determine the functional activity of the CP.

#### 3.1.2 Measurement of individual complement proteins

In Basel, levels of C3 and C4 were determined using nephelometry. For the C3 measurement, a polyclonal anti-C3c antibody was used. This antibody was raised against the recombinant human C3c fragment and is reactive to both C3 and its respective fragment. C2 levels were assessed using turbidimetry. In Lund/SE, the levels of C1 inhibitor were measured by nephelometry. The levels of C1q, C1r, and C1s were measured by electroimmunoassay (“rocket” immunoelectrophoresis) and expressed in percentage of the concentration in a standard serum pool consisting of sera from healthy blood donors: 50 men and 50 women (18, 19).

#### 3.1.3 Functional complement assays and C1s reconstitution

To obtain serum, blood samples were allowed to clot for 1 h and then centrifuged at 1,200×*g* for 15 min at room temperature (RT), and the serum was immediately stored at −70°C. Human IgM (Bio-Rad, Hercules, CA, USA) was coated overnight at 4°C, at 2 µg/ml in 100 µl 0.1 M carbonate buffer (pH 9.6) in MaxiSorp microplates (Thermo Fisher, Waltham, MA, USA) for activation of the CP (20). If not stated differently, all further incubation steps were conducted for 1 h at RT on a rocking plate at 300–400 rpm, and plates were washed three times with a washing buffer (PBS + 0.05% Tween) after each incubation step. After coating and washing, the plates were blocked with 200 µl of PBS (Sigma-Aldrich, St. Louis, MO, USA) + 1% BSA (Sigma-Aldrich). Serum samples were centrifuged at 14,000×*g* for 30 min at 4°C before use. In the case of C1s reconstitution, the samples were incubated for 30 min on RT with 33 μg/ml of C1s proenzyme (Complement Technologies, Tyler, TX, USA) since the normal adult concentration of C1s was found to be 32.8 ± 6.2 µg/ml (1 SD) (21). Serum samples were then diluted 1:100, either in HBS (HEPES-buffered saline) buffer or in PBS + 0.05% Tween + 10 mM EDTA. Samples were added in duplicates to the plate (100 µl/well) and incubated for 1 h at 37°C. To determine whether activation of the CP had taken place, rabbit anti-human C4d (Abcam, Cambridge, UK) was used, which was diluted 1:400 in PBS + 0.05% Tween and added in a volume of 100 µl/well. Plate-bound anti-C4d antibody was detected by incubation with 100 µl of HRP-conjugated goat anti-rabbit IgG (Santa Cruz Biotechnology, Dallas, TX, USA), which was diluted in 1:4,000 PBS + 0.05% Tween. After five times washing, color development with TMB substrate (BD Headquarters, Franklin Lakes, NJ, USA) was performed as recommended by the manufacturer. The reaction was stopped after 20 min by the addition of 100 µl 0.5 M H_2_SO_4_, and color development was read at 450 nm.

#### 3.1.4 Whole genome sequencing

Whole genome sequencing of germline nuclear DNA was performed at the Department of Clinical Genetics and Pathology, Laboratory Medicine, Region Skåne, Lund, Sweden, using TruSeq DNA PCR-Free (Illumina, San Diego, CA, USA) and NovaSeq 6000 (Illumina). Reads were aligned to the human genome with GRCh38 as the reference using the Burrows–Wheeler Aligner software (SourceForge, CA, USA). Further data analysis was performed with Sentieon software (Sentieon, Mountain View, CA, USA). The procedure selects SNVs and indels in exons and 20 bp of flanking introns, as well as other intron variants previously reported in ClinVar, while clinically relevant CNVs are reported from the whole genome. A targeted gene panel was used, comprising 42 genes related to the complement system. Variant interpretation was performed in Scout software (22) by a molecular geneticist and verified by a clinical immunologist (LS).

### 3.2 Results

#### 3.2.1 Complement protein levels

The patient’s complement profile is shown in **Table 1**. No hemolytic activity mediated by the CP was detected in the deficient sera. The AP had normal function.

An estimation of C1s by rocket immunoelectrophoresis showed no detectable C1s. C1q and C1r were low at 40% and 23% of normal in serum, respectively. The levels of C3 as determined by nephelometry were in a normal range (0.96 g/L), whereas C4 (0.44 g/L), C2 (Lund 164% of normal, Basel 0.044 g/L), and C1 inhibitor (0.63 g/L) were above the reference.

#### 3.2.2 C1s reconstitution

After IgM coating and the addition of serum, C4d deposition on the plate was measured by ELISA as a marker for activation of the classical pathway. The results are shown in **Figure 3**. Measurement of complement activation in EDTA was used as a negative control, as well as the sera of C1q-deficient patients. The sera of healthy blood donors showed complement C4d deposition in HBS buffer with a decrease after the addition of C1s proenzyme. The serum of the patient showed no complement activation without reconstitution but had strong C4d deposition after reconstitution with C1s proenzyme exceeding the one observed for NHS. As an additional control, the sera of two C1q-deficient patients showed no activation of the CP as judged by C4d deposition, independent of the addition of C1s.

#### 3.2.3 Characterization of the genetic defect

One variant was located at nucleotide 514 in exon 5 of the *C1S* gene, caused by a nucleotide substitution from guanine to thymine, resulting in a nonsense mutation at codon 172. The other variant was located at nucleotide 750 in exon 7 of the *C1S* gene, where cytosine was replaced by guanine, resulting in a nonsense mutation at codon 250. Both mutations create a premature stop codon and have not previously been reported in the literature. There were no significant variants in the *C1qA*, *B*, or *C* genes or in the *C1R* gene and no other relevant variants in complement genes.

## 4 Discussion

We identified a compound heterozygous genotype of the *C1S* gene in our patient, manifesting as a selective C1s deficiency. Notably, the detected variants p.Gly172* in exon 5 and p.Tyr250* in exon 7 have not previously been reported in the literature. C1s deficiency is inherited in an autosomal recessive mode (9–11). However, since no relatives were accessible for genetic analysis, we were not able to prove that the variants are in transposition.

This patient’s history of recurrent severe infections is based on the crucial role of C1s in activating the CP (23, 24). The diagnosis of chilblain lupus in his adolescence, which later progressed into SLE with severe kidney involvement, aligns with documented cases suggesting that deficiency in early components of the CP predisposes to severe cutaneous lesions and renal disease. Additionally, these patients have a low incidence of anti-dsDNA (4), which was also the case in our patient, whose anti-dsDNA was only one time weakly positive in the context of a severe flare.

Functional complement assays revealed unmeasurably low CH50 activity in our patient, hinting at a complement deficiency. Consistent with other C1s-deficient individuals, the levels of C1q and C1r were decreased, probably due to increased consumption in non-complexed form (12). Although classical SLE patients often exhibit reduced levels of C4, C2, and C3, our patient’s increased levels of these complement proteins, in conjunction with C1 inhibitor, mirror the findings as described in other C1r/C1s-deficient individuals (10, 11). In the absence of C1s, the CP cannot be activated and the consumption of the subsequent complement proteins is reduced. In contrast to previous reports (11), we were able to restore complement activation through the CP in the patient’s serum by the addition of C1s proenzyme. This finding, together with the gene sequencing results, suggests an isolated absence of C1s in our patient.

Renal biopsy findings were also notable. While strong C1q and C5b-9 deposition aligns with typical LN (25), the weak deposits of C3, C4, IgG, and IgM stand out. However, multiple factors may have influenced the staining intensities, with a relatively high affinity of the antibody used for C5b-9 maybe accounting for the observed discrepancy between stainings for C3 and C5b-9. The mechanism by which deficiencies of the early CP lead to the development of SLE and LN has not yet been elucidated (13). Especially intriguing is the presentation of LN in the absence of anti-C1q antibodies, which is atypical (26, 27). In addition, weak renal deposits and, in particular, the lack of peripheral consumption of C3 and C4 suggest a rather minor pathogenic role of the LP and AP in our patient.

Based on the observation that there is a hierarchy of disease susceptibility according to the position of the missing protein in the activation pathway, increasing from C2 over C1r/s and C4 to C1q deficiency (4, 28), we propose the cumulative deficiency hypothesis. Assuming that each molecule of the early CP has a protective function, the observed hierarchical arrangement might be an expression of a cumulative (functional) deficiency. This hypothesis implies that the earlier the deficient protein is located within the pathway, the more pronounced the cumulative effect of its absence will be on the activation of downstream complement components. For example, the absence of C1q would compromise the entire classical pathway and consequently lead to the highest susceptibility to SLE. In comparison, the absence of C1s or C1r also compromises the classical pathway early on, but at least C1q can still deposit. Theoretically, the absence of C1r or C1s consequently would result in a similar level of defective classical pathway activation as a lack of C4, meaning no activation of C4 and C2 via this pathway, but the penetrance of SLE would be lower than in C1q deficiency. However, this hypothesis, while intriguing, necessitates further validation.

Last, the administration of rituximab proved to be effective in our patient. Rituximab is a chimeric monoclonal antibody targeting CD20 on B cells leading to the depletion of mature B cells and their precursors, but not plasma cells, which lack CD20 expression (29). In SLE, B cells can drive autoimmune responses beyond just antibody production, such as presenting autoantigens to T cells and producing cytokines that affect other immune cells (30–32).

The success of rituximab in our patient, who was negative for specific autoantibodies like anti-C1q and anti-ds-DNA, which are believed to be implicated in the pathogenesis of SLE, emphasizes the central importance of B cells in SLE, even beyond their capacity as autoantibody producers. However, the future course of the disease in our patient with C1s deficiency needs to show how sustained the efficacy of a treatment with rituximab in this setting will be.

In conclusion, this rare presentation of C1s deficiency associated with LN highlights open questions about the role of the CP in the etiopathogenesis of SLE.

## Data availability statement

The datasets presented in this article are not readily available due to legal and ethical restrictions related to the protection of patient data and privacy. The Whole Genome Sequencing data is subject to special protections and cannot be made publicly available. Requests to access the datasets should be directed to Jessica S. Kleer.

## Ethics statement

Written informed consent was obtained from the individual(s) for the publication of any potentially identifiable images or data included in this article.

## Author contributions

JK: Visualization, Writing – original draft, Software, Data curation, Formal Analysis. LS: Data curation, Writing – original draft. DD: Investigation, Writing – original draft. IF: Investigation, Writing – original draft. AZ: Investigation, Writing – original draft. EM: Investigation, Writing – original draft. MK: Investigation, Supervision, Writing – review & editing. MT: Conceptualization, Funding acquisition, Methodology, Project administration, Resources, Supervision, Writing – review & editing.
